# Placement of a drainage tube in the ileac lumen following laparoscopic appendectomy: A case report and literature review

**DOI:** 10.1097/MD.0000000000038405

**Published:** 2024-06-07

**Authors:** Wei Dong, Meng Qiu, Xuhui Ma, Shunchang Zhou, Hao Chen, Haibo Chu, Yuxu Zhong

**Affiliations:** a Department of General Surgery, Jiaozhou Branch of Shanghai East Hospital, Tongji University, Qingdao, China; b State Key Laboratory of Toxicology and Medical Countermeasures, Institute of Pharmacology and Toxicology, Beijing, China.

**Keywords:** drainage tube, Ileac perforation, laparoscopic appendectomy

## Abstract

**Rationale:**

Ileal perforation caused by the insertion of a drainage tube is a rare complication. Hence, the utilization of surgical drains in abdominal surgery remains controversial. At present, there is a trend to reduce the utilization of drains in abdominal surgery, although certain situations may necessitate their application.

**Patient concerns:**

A 25-year-old Chinese woman presented with a history of right lower abdominal pain persisting for 10 days. Imaging examinations, including abdominal computed tomography and ultrasound, identified low-density lesions measuring 10 × 8 × 8cm^3^ in the right lower abdomen, which are consistent with perforated appendicitis complicated by a peri-appendiceal abscess. A laparoscopic appendectomy was carried out. On the 5th postoperative day, the drainage fluid changed to a grass-green color (80mL). Imaging with retrograde contrast through the drainage tube revealed that the 26 Fr silicon rubber drainage tube tip was positioned 50cm away from the ileocecal junction within the ileum. Both the ileal and ileocecal regions appeared well-developed.

**Intervention and outcomes:**

Oral intake was suspended, and the patient received antacids, somatostatin, antibiotics, and total parenteral nutrition. On the 19th postoperative day, a follow-up imaging procedure using retrograde contrast through the drainage tube indicated that the tube tip was sealed. The treatment concluded on day 33 postoperatively, and the patient was discharged.

**Discussion and conclusion:**

Ileal perforation due to an abdominal drainage tube following laparoscopic appendectomy constitutes a rare but serious complication. However, due to the adhesion and inflammatory changes around the abscess, laparoscopic dissection becomes a challenging and risky process, and the surgical skills and experiences are particularly important. Removing the abdominal drainage tube promptly based on the characteristics of the drainage fluid is recommended. The findings provide valuable insights for surgeons navigating similar challenges.

## 1. Introduction

Drainage tubes have been widely utilized to provide various benefits, including the reduction of postoperative infections and the prevention of fluid or air accumulation.^[1]^ The utilization of surgical drains in abdominal surgery remains a subject of controversy. Although their role is acknowledged in specific circumstances, their systematic usage endorsed by some clinicians remains contentious.^[2]^ As Dr Sims first applied abdominal drainage tubes for patients undergoing gynecological surgery in the 1870s, surgeons have extensively employed them in clinical practice.^[3]^ The results of a meta-analysis indicated that routine abdominal drainage increases the incidence of postoperative biliary leakage, particularly following drainage removal.^[4]^ However, complications, such as digestive tract perforation resulting from the insertion of a drainage tube have been reported.^[5]^ Notably, ileal perforation due to an abdominal drainage tube represents an unreported complication in this context. This study aimed to present a case of ileal perforation following laparoscopic appendectomy in a patient with a peri-appendiceal abscess.

## 2. Case presentation

**Figure 1.**
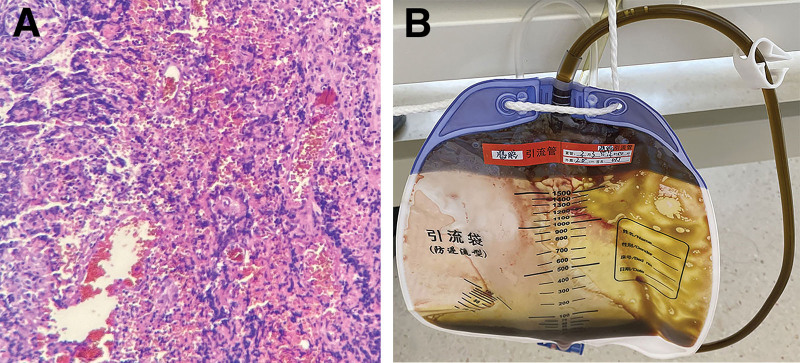
(A) Histological examination of the resected specimen revealed the infiltration of many neutrophils into the whole layer of the appendix, exudated fibrin, and focal necrosis (hematoxylin–eosin staining, magnification × 100). (B) On the 5th postoperative day, the patient reported the drainage of 30 mL of grass-green fluid from the abdominal cavity everyday.

A 25-year-old Chinese woman presented with a history of right lower abdominal pain persisting for 10 days. She reported an absence of fever and diarrhea. Her overall health was otherwise unremarkable, and she weighed 60 kg. Physical examination revealed a body temperature of 36.8 °C and tenderness localized to the right lower quadrant. Laboratory tests indicated a white blood cell count of 12.4 × 10^9^/L and a neutrophil rate of 92%. Imaging examinations, including abdominal computed tomography (CT) and ultrasound, identified low-density lesions measuring 10 × 8 × 8 cm^3^ in the right lower abdomen, which are consistent with perforated appendicitis complicated by a peri-appendiceal abscess. The appendix was intraoperatively found in a retrocecal position, with evidence of perforation at the root. The area was also wrapped by the omentum and adjacent intestine, measuring 10 × 8 × 8 cm^3^. Additionally, a free fecalith with a diameter of 1 cm was discovered along with 350 mL of pus in the peri-appendiceal abscess and 100 mL in the pelvic cavity. Laparoscopic appendectomy was carried out, and postoperative pathology confirmed the diagnosis of purulent appendicitis (Fig. 1A).

**Figure 2.**
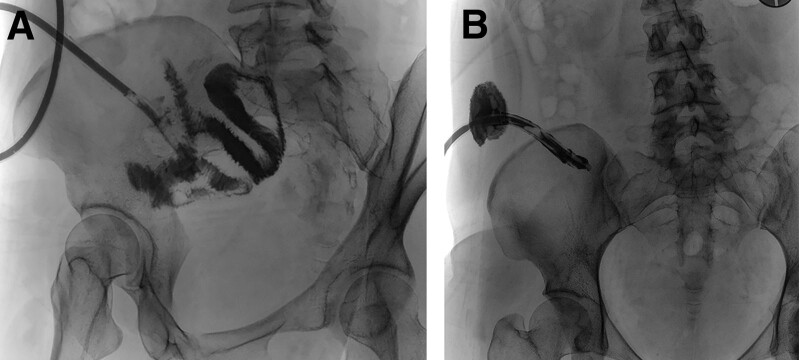
(A) Retrograde imaging of the drainage tube demonstrated that the drainage tube tip was located 50-cm ileum away from the ileocecal part. The ilealand ileocecal parts were developed. (B) Retrograde imaging of the drainage tube showed that the drainage tube tip was closed, with no contrast agent into the proximal ileum.

**Figure 3.**
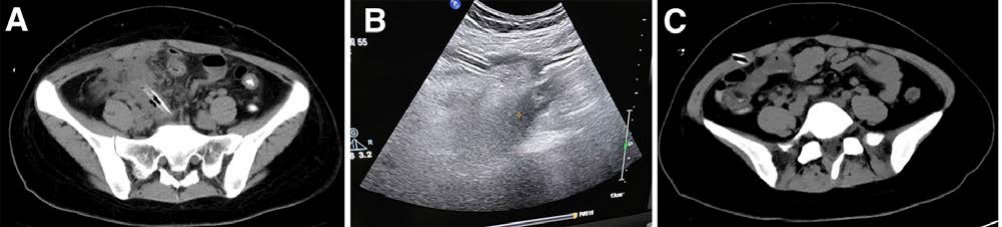
(A) Abdominal computed tomography showed no encapsulated effusion in the right lower abdomen. (B) Ultrasound showed a hypoechoic lesion with diameters 0.8 × 1.0 cm^2^ in the surrounding drainage tube of the abdomen after withdrawing the drainage tube. (C) Abdominal computed tomography indicated that the drainage tube tip was not in situ and no encapsulated effusion occurred in the abdominal cavity.

On the 2nd postoperative day, anal exhaust and defecation were noted, and the drainage fluid was quantified at 30 mL per day, exhibiting a gray-yellow appearance. On the 5th postoperative day, the patient experienced neither fever nor abdominal symptoms, and the drainage fluid changed to a grass-green color (80 mL), as illustrated in Figure 1B. Imaging with retrograde contrast through the drainage tube revealed that the 26 Fr silicon rubber drainage tube tip was positioned 50 cm away from the ileocecal junction within the ileum. Both the ileal and ileocecal regions appeared well-developed, and no leakage of the contrast agent was found at the appendiceal stump (Fig. 2A). Abdominal CT scan confirmed the absence of encapsulated effusion in the right lower quadrant (Fig. 3A). Oral intake was suspended, and the patient received antacids, somatostatin, antibiotics, and total parenteral nutrition (TPN). Over the subsequent 9 days, the grass-green drainage fluid volume ranged from 50 to 100 mL per 24-h. Dynamic ultrasonography did not reveal any encapsulated effusion. On the fourteenth postoperative day, following another imaging procedure using retrograde contrast through the drainage tube, the tube was retracted by 6 cm and its tip was found to be outside the intestinal lumen. Following tube retraction, intermittent fevers, fluctuating between 37.6 and 38.9 °C, were managed with antipyretic medications. The patient reported no abdominal discomfort, and hematological parameters remained within normal ranges. Follow-up ultrasound imaging identified a cord-like hypoechoic area in the right lower abdomen, measuring approximately 0.8 × 1.0 cm^2^ (Fig. 3B). On the 19th postoperative day, a follow-up imaging procedure using retrograde contrast through the drainage tube indicated that the tube tip was sealed; no contrast agent was found entering the proximal ileum or leaking into the abdominal cavity (Fig. 2B). Abdominal CT scan corroborated that the drainage tube tip was no longer in its original position and no encapsulated effusion was present (Fig. 3C). The drainage tube was removed on the same day, and enteral nutrition (EN) was initiated. After 2 weeks of enteral feeding, follow-up contrast-enhanced abdominal CT scan displayed no abnormality. The treatment concluded on day 33 postoperatively, and the patient was discharged.

## 3. Discussion

Ding et al^[6]^ reported a patient who underwent cholecystectomy, and bile-stained fluids were extracted from the drainage tube on the 6th postoperative day. Imaging with retrograde contrast through the drainage tube showed duodenal imaging, confirming the presence of a duodenal fistula. In a study conducted by Eleftheriadis^[5]^ on the 4th day after total gastrectomy for gastric cancer, bile-stained fluids were extracted from the drainage tube, and the gastroscopy indicated that the tip of the drainage tube was located in the jejunum, and on the 6th postoperative day for hepatic echinococcosis, bile-stained fluids were extracted from the drainage tube, reaching up to 800 mL within 24 hours. The gastroscopy revealed that the tip of the drainage tube was located in the stomach cavity. Shao et al^[7]^ reported a patient who underwent splenectomy due to traumatic splenic rupture. On the seventh postoperative day, the drainage tube removed 50 mL of purulent fluid. On the 9th postoperative day, 500 mL of bile-colored fluid with small food particles was found in the drainage tube. A similar study conducted by Paraskevopoulos et al^[8]^ concentrated on splenectomy following traumatic spleen rupture. On the 6th postoperative day, the drainage tube started releasing an increasing volume of bile-stained fluid, as confirmed by a gastrografin study (administered through the nasogastric tube), revealing that the drainage tube was situated at a higher position along the greater curve of the stomach. Sato et al^[9]^ reported a patient who underwent laparoscopic low anterior resection for rectal cancer, during which a transanal decompression tube (24 Fr Nelaton catheter) was laparoscopically placed. A contrast enema on the 5th postoperative day indicated perforation of the sigmoid colon around the tip of the tube, leading to emergency laparotomy. Similarly, Hiraki et al^[10]^ reported a case of perforation of the anterior wall of the proximal colon due to transanal decompression tube placement. Analysis of these studies revealed that digestive tract perforations caused by drainage tubes predominantly occurred between the fourth and seventh postoperative days, with the highest frequency observed on days 5 to 6. Perforations at high positions (e.g., stomach, duodenum, and jejunum) were associated with significant drainage fluid (500–800 mL/24 hours), whereas lower position perforations (e.g., ileum and colon) were less common.^[5,7]^ The most direct method to determine perforation caused by drainage tube is gastroenteroscopy or imaging with retrograde contrast through the drainage tube.

In the present case, on the 14th postoperative day, it was attempted to withdraw the tip of the drain from the ileal wall, as guided by X-ray imaging. Following the withdrawal of the drainage tube, the patient exhibited intermittent fever, while she did not experience abdominal pain or distension. Laboratory tests confirmed a normal hemogram. Ultrasound imaging revealed a cord-like hypoechoic area in the right lower abdomen. Given these observations, it could be concluded that the fistula exhibited a low output, suggesting a likelihood of spontaneous healing. To assess the situation more comprehensively, dynamic imaging was conducted through both ultrasound and CT scan over a 3-day period. The results indicated no increase in the outflow of ileal fluid around the tip of the drainage tube. Additionally, imaging confirmed that the tip of the drainage tube was sealed, as no contrast agent entered the proximal ileum, suggesting that the fistula had spontaneously been healed. We hypothesized that any rise in the outflow of ileal fluid around the drain tube tip would require intervention. In such cases, replacing the double cannula leach with X-ray guidance and initiating continuous flushing with negative pressure drainage were suggested to accelerate fistula healing. Neglecting increased outflow could lead to the prompt development of an abdominal abscess.

Somatostatin serves as a natural hormone that regulates a variety of physiological functions, including the inhibition of insulin and glucagon secretion, as well as the modulation of gastric, duodenal, and gallbladder motilities, and the secretion of intestinal and gastric juices. Historically, the gold standard for treating fistulae, such as pancreatic and enterocutaneous types, has involved the utilization of TPN in conjunction with a continuous infusion of somatostatin. This regimen typically includes the intravenous administration of TPN, comprising carbohydrates, lipids, amino acids, vitamins, and oligoelements, alongside somatostatin. However, the efficacy of adding somatostatin directly to the TPN mixture for promoting fistula closure still requires further comparison with the conventional infusion methods.^[11]^ In our clinical experience treating intestinal fistulas, upon confirmation of an intestinal fistula, intravenous administration of somatostatin (1.2 mg) with normal saline (40 mL) and omeprazole (80 mg) with normal saline (40 mL) can be continuously infused for 24 hours, to minimize the amount of digestive fluid overflow in the abdominal cavity, thereby reducing the incidence of abdominal infection and sepsis. Furthermore, antibiotics were administered to control abdominal infection. Abdominal CT scan was carried out to monitor fluid accumulation in the abdominal cavity. In the event of intraperitoneal encapsulated effusion, Lee double cannula was placed in the abdominal cavity under ultrasound guidance for continuous abdominal irrigation and negative pressure drainage.

EN has emerged as an alternative treatment modality to maintain the patient’s nutritional requirements and preserve intestinal mucosal integrity. Recent studies have demonstrated that EN enhances the gastrointestinal tract’s immunity, reduces infection risk, diminishes fistula output, and elevates the likelihood of fistula closure (60% vs 37%) compared with immunonutrition protocols. Nevertheless, EN does not significantly influence mortality rate.^[11]^ EN’s advantages include the maintenance of the intestinal mucosal barrier and its immunological functions, as well as the stimulation of intestinal trophic hormones, such as growth hormone, epidermal growth factor, enteroglucagon, and insulin-like growth factor I.^[12]^ After recovery of intestinal function, abdominal CT scan displayed no effusion, the doses of somatostatin and omeprazole were reduced and their administration was then discontinued, and EN was immediately commenced. By observing the clarity of abdominal drainage fluid and retrograde-enhanced angiography of the drainage tube, the healing of the fistula was confirmed, and continuous irrigation and negative pressure drainage were conducted, regardless of the situation. Once the sinus had formed, it could be gradually removed or replaced with a slender drainage tube (red rubber catheter), using contrast media and rubber tube stimulation to facilitate sinus closure.

To our knowledge, the penetration of the ileus by a drainage tube has not been previously reported. However, the appropriate selection of tube, including its size, materials, site, length of placement, and duration of application, remains a topic of debate. In the majority of studies, silicone rubber tubes were utilized, and the tube sizes typically ranged from 24 to 32 Fr (approximately 8–10 mm),^[13,14]^ while Lee et al^[15]^ demonstrated that perforation occurred in a 10 Fr small diameter tube. It was found that the perforation of digestive tract caused by drainage tube has no correlation with its thickness. Luo et al^[16]^ concluded that the utilization of a rigid drain through soft tissues unaccustomed to pressure might result in pressure necrosis of adjacent tissues. Contributing factors may include pressure exerted by intraluminal contents and edema formation at the site of surgical manipulation. Recent advancements in both the materials and design of drainage tubes have rendered these devices less traumatic for patients.

At present, laparoscopic surgery is widely utilized in the treatment of complex appendicitis, including the drainage of peri-appendiceal abscesses.^[17,18]^ However, due to the adhesion and inflammatory changes around the abscess, laparoscopic dissection becomes a challenging and risky process, and the surgical skills and experiences are particularly important. Although there is controversy over the placement of drainage tubes, there is a tendency to use less drains in abdominal surgery.^[2]^ In this case, varying degrees of serous layer damage might occur during the intestinal separation procedure, leading to short-term inflammatory edema of the intestinal wall. Following the insertion of a robust silicone rubber drainage tube (26 Fr), there would be a likelihood of the drainage tube penetrating into the intestinal lumen. It was noted that the patient’s drainage of intestinal fluid was <100 mL/day, and there was no fluid accumulation in the abdominal cavity. This indicates that the distal end of the injured bowel was unobstructed and the drainage tube was wrapped by an inflammatory fibrous tissue. After half a month, the damaged intestinal wall can thicken. Upon the drainage tube exiting the bowel, the rapid formation of hard fibrous tissue may prompt closure. In addition, there is no gap in the inflammatory adhesion between the intestinal walls, which will not cause intestinal fluid to penetrate the abdominal cavity. When placing coarse and rigid drainage tubes for patients with intestinal wall injuries, it is imperative to contemplate early extubation based on the nature and volume of drainage fluid to prevent potential complications, such as intestinal perforation or obstruction induced by the drainage tubes.^[1,4]^ Aligning with this perspective, Durai et al^[19]^ suggested that the removal of a drainage tube could be considered relatively safe if the drainage flow remained below 25 mL within 24 hours.

## 4. Conclusions

In conclusion, ileal perforation due to an abdominal drainage tube following laparoscopic appendectomy constitutes a rare while serious complication. To mitigate this risk, the following measures are recommended: opt for a soft drainage tube, paying further attention to its length; position the drainage tube in the right iliac fossa to the greatest extent possible, avoiding contact with inflamed bowel; remove the abdominal drainage tube promptly based on the characteristics of the drainage fluid. In managing intestinal fistulas, clinicians should employ a combination of withheld oral intake, antacids, somatostatin, antibiotics, and TPN at 2000 calories per day. In the present case, the drainage tube was removed half a month post-surgery, as guided by imaging, thereby minimizing the risk of intestinal fluid spillage and abdominal abscess formation. Following tube removal, the patient transitioned to enteral feeding. The follow-up contrast-enhanced abdominal CT scan conducted 2 weeks later confirmed complete fistula healing. The findings provide valuable insights for surgeons navigating similar challenges.

## Acknowledgments

We would like to thank Prof Yang Qing, Department of Pathology in Jiaozhou Branch of East Hospital, Tongji University for our guidance and help to us in the pathology of this study.

## Author contributions

**Investigation:** Haibo Chu, Wei Dong, Meng Qiu.

**Methodology:** Haibo Chu.

**Project administration:** Haibo Chu.

**Writing – review & editing:** Haibo Chu, Yuxu Zhong.

**Writing – original draft:** Wei Dong, Meng Qiu.

**Data curation:** Xuhui Ma, Shunchang Zhou.

**Formal analysis:** Shunchang Zhou, Hao Chen.

**Funding acquisition:** Yuxu Zhong, Wei Dong.
